# Evidence for redox sensing by a human cardiac calcium channel

**DOI:** 10.1038/srep19067

**Published:** 2016-01-11

**Authors:** Padmapriya Muralidharan, Henrietta Cserne Szappanos, Evan Ingley, Livia Hool

**Affiliations:** 1 School of Anatomy, Physiology and Human Biology, The University of Western Australia, Crawley, WA, Australia; 2 Harry Perkins Institute of Medical Research, QEII Medical Centre, Nedlands and Centre for Medical Research, The University of Western Australia, Crawley, WA, Australia; 3 Victor Chang Cardiac Research Institute, Sydney, NSW, Australia

## Abstract

Ion channels are critical to life and respond rapidly to stimuli to evoke physiological responses. Calcium influx into heart muscle occurs through the ion conducting α1C subunit (Ca_v_1.2) of the L-type Ca^2+^ channel. Glutathionylation of Ca_v_1.2 results in increased calcium influx and is evident in ischemic human heart. However controversy exists as to whether direct modification of Ca_v_1.2 is responsible for altered function. We directly assessed the function of purified human Ca_v_1.2 in proteoliposomes. Truncation of the C terminus and mutation of cysteines in the N terminal region and cytoplasmic loop III-IV linker did not alter the effects of thiol modifying agents on open probability of the channel. However mutation of cysteines in cytoplasmic loop I-II linker altered open probability and protein folding assessed by thermal shift assay. We find that C543 confers sensitivity of Ca_v_1.2 to oxidative stress and is sufficient to modify channel function and posttranslational folding. Our data provide direct evidence for the calcium channel as a redox sensor that facilitates rapid physiological responses.

Ischemic heart disease is the leading cause of death in the western world^1^. Myocardial infarction or heart attack occurs as a result of coronary artery occlusion and involves deprivation of oxygen (hypoxia) and blood flow leading to necrosis of the affected area of muscle. Reperfusion of heart muscle is the primary clinical objective to salvage myocardium but paradoxically this causes an increase in oxygen and the generation of reactive oxygen species that further injures the myocardium^2^. Reperfusion injury involves alterations in calcium homeostasis and increases in reactive oxygen species that participate in the progression of pathology by activating growth promoting signalling pathways. This leads to the development of cardiac hypertrophy and failure^3^,^4^. One of these reactive oxygen species, hydrogen peroxide (H_2_O_2_), interacts with cell signalling pathways by way of modification of key thiol groups on proteins that possess regulatory functions^5^. H_2_O_2_ is a small, stable molecule and able to freely cross membranes. It is also capable of oxidizing the thiol groups of cysteine residues to form a disulfide bond with glutathione (GSH) (glutathionylation). The functional implications of glutathionylation are not well understood but are becoming increasingly recognised in disease.

The L-type calcium channel is critical to cardiac function and the main route for calcium influx into cardiac myocytes^6^. Ca^2+^ influx through the L-type Ca^2+^ channel shapes the long plateau phase of the ventricular action potential and initiates the sequence of events that result in contraction^7^. Cardiac L-type Ca^2+^ channels are heterotetrameric polypeptide complexes consisting of α_1C_, α_2_δ and β_2_ subunits. Cardiac muscle expresses the dihydropyridine sensitive α_1C_ subunit, which is encoded by the CACNA1C gene and known as Ca_v_1.2. The Ca_v_1.2 is the main pore forming subunit and is sufficient for calcium conductance. The auxiliary β_2_ subunit binds to the I-II linker in the Ca_v_1.2 subunit at the alpha interacting domain and facilitates the trafficking and insertion of the polypeptide complex into the membrane^8^,^9^. The α_2_δ and β_2_ subunits modify the inactivation kinetics of the current^8^,^9^,^10^.

It is well recognized that the function of the native L-type Ca^2+^ channel can be regulated by hypoxia and oxidative stress^11^,^12^,^13^,^14^. It has been proposed that the Ca_v_1.2 protein contains an “oxygen sensing region” encoded by exon 45^15^. However, a direct effect of hypoxia on Ca_v_1.2 function has not been demonstrated^16^. In addition, thiol reducing agents have been demonstrated to mimic the effects of hypoxia on Ca^2+^ currents while thiol oxidising agents increase Ca^2+^ currents or attenuate the effects of hypoxia^13^,^14^,^17^,^18^,^19^,^20^,^21^,^22^,^23^,^24^. This has led to the proposal that the channel is not the oxygen sensor but can respond to alterations in the cell’s redox state. In addition, Ca_v_1.2 protein is glutathionylated in guinea pig myocytes after ischemia reperfusion injury and in ischemic human heart^25^. Glutathionylation of Ca_v_1.2 causes an increase in calcium influx^25^. This suggests that direct glutathionylation of the channel may regulate function during oxidative stress.

The channel protein contains many cysteines that are available to be covalently modified during changes in redox state. However, it is not clear how modification of thiols alters channel function because contradictory responses have been reported, with some groups arguing that thiol reducing agents increase channel activity while others argue the opposite response^26^. The variation in functional responses could be a result of the influence of a signalling protein in the cellular environment, or an accessory protein or subunit that modifies channel function. It is also not clear whether direct reduction or oxidation of thiol groups on the channel protein is sufficient to alter function. In this study we reconstituted purified human Ca_v_1.2 in liposomes for functional assessment. Under these conditions, only channel protein and lipid are present. We find that the open probability of the channel is altered when exposed to thiol oxidising or reducing agents. Truncating the C-terminal domain of the protein further increased the open probability of the channel but did not alter the response to thiol reducing and oxidising agents. We find that mutation of three critical cysteines in the loop I-II linker region of the channel prevents the functional response of the channel to thiol modification and alters protein folding. Finally we demonstrate that mutation of C543S alone is sufficient to attenuate the effect of thiol modifying agents. Our results indicate that direct modification of the Ca_v_1.2 channel protein is responsible and sufficient for alterations in channel function during oxidative stress.

## Results

### Characterisation of single channel currents from purified Ca_v_1.2 channel protein

**Figure 1:**
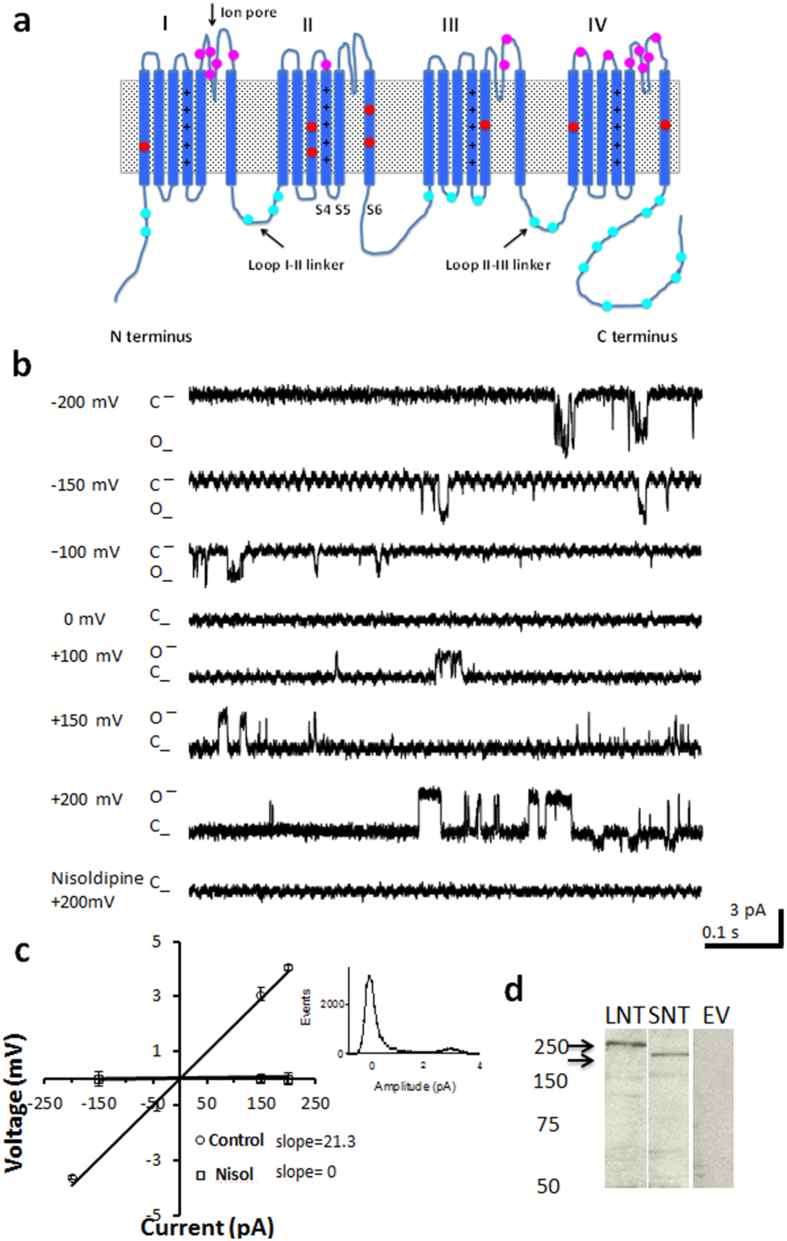
Characterisation of the Ca_v_1.2 current of the human long NT isoform reconstituted in liposomes. (**a**) Topology of the Ca_v_1.2 channel protein indicating the voltage sensing transmembrane spanning region S4 and pore forming (S5-S6) regions (Ion pore) and cytoplasmic linker regions. Cysteines are shown as circles in cytoplasmic, transmembrane and extracellular regions. Mutant constructs were generated in the N-terminus (NT), loop I-II and Loop III-IV regions (for further details see Results and Methods sections). Double lines indicate where the C-terminus was truncated. (**b**) Representative single-channel currents recorded at varying pipette potentials as indicated. 2 μM nisoldipine (Nisol) abolished channel activity in the patch. (**c**) Current-voltage (I–V) relationship recorded from the same patch in the absence and presence of nisoldipine. A current-amplitude frequency histogram constructed from 20 s of single-channel data recorded at + 100 mV is shown at right. (**d**) Immunoblot of the long NT isoform of the Ca_v_1.2 channel protein indicated at approximately 240 kDa when probed with anti-Ca_v_1.2 antibody (LNT), the short NT isoform of the Ca_v_1.2 channel protein indicated at approximately 220 kDa when probed with anti-Ca_v_1.2 antibody (SNT) and protein from HEK293 cells expressing pcDNA3.1 plasmid minus Ca_v_1.2-His (Empty vector = EV).

The Ca_v_1.2 channel is a large protein approximately 240 kDa comprised of 4 repeating domains (I-IV) (Fig. 1a,d). Each domain contains a voltage sensing region in transmembrane spanning segment S4 and the pore forming ion conducting region in S5-S6. When reconstituted in liposomes the Ca_v_1.2 alpha subunit forms a functional ion conducting protein. For each experiment we confirmed the properties of the channel by voltage-stepping the channel in symmetric solution from –200 mV to +200 mV and measuring the magnitude of the current and the sensitivity of the current to the L-type calcium channel antagonist nisoldipine. We used the same solution in the bath and patch-pipette that composed of BaCl_2_, NaCl and the dihydropyridine agonist BayK(–) to promote channel openings. Voltage stepping the long NT isoform protein to –200 mV elicited an inward current approximately –4 pA in magnitude that decreased as voltage approached 0 mV (Fig. 1b) reversed at 0 mV (consistent with symmetric solutions) with a slope conductance of approximately 21.3 pS, similar to that previously reported in bovine calcium channels^27^. The current was attenuated in the presence of nisoldipine. The channel protein was confirmed by immunoblot analysis (Fig. 1c). The open probability of the channel (P_o_) was 0.020 ± 0.003 (n = 33) and the mean open dwell time was approximately 18.6 ± 2.4 ms (n = 13), consistent with long channel openings associated with binding by BayK(–).

### Short and Long NT isoforms respond similarly to thiol reducing and oxidising agents

**Figure 2:**
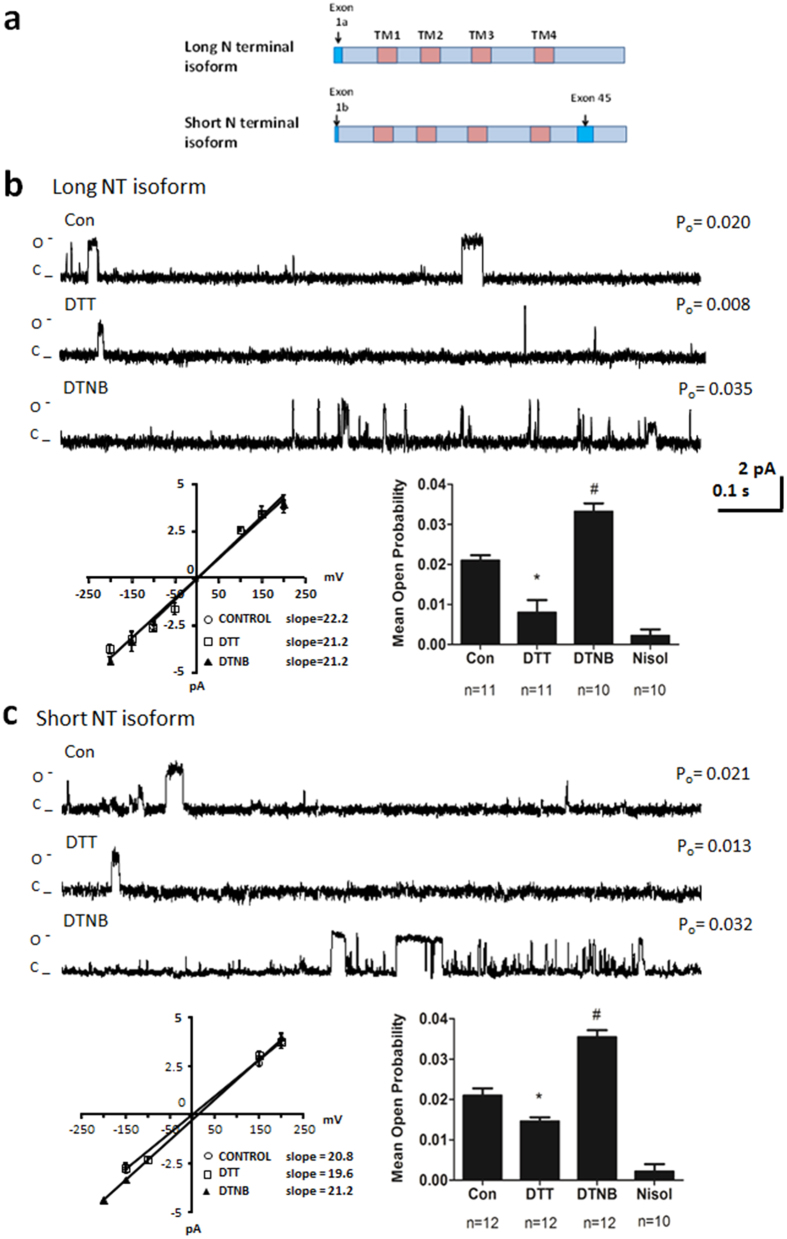
The long NT isoform and short NT isoform respond similarly to DTT and DTNB. (**a**) Schematic of long and short NT isoforms indicating transmembrane spanning regions (TM) and variable exons. (**b**) Representative single-channel currents of the long NT isoform recorded at + 100 mV in the absence and presence of 1 mM DTT followed by 200 μM DTNB in the same patch as shown. The I–V relationship (below left) and the mean ± SEM channel open probability (P_o_) for currents recorded in control solution and currents recorded in the presence of DTT, DTNB and nisoldipine (Nisol) are shown below right. ^#,^*p < 0.05 vs Con ANOVA and Tukey’s Posthoc test (**c**) Representative single-channel currents of the short NT isoform recorded at + 100 mV in the absence and presence of 1 mM DTT followed by 200 μM DTNB in the same patch as shown. The I–V relationship (below left) and the mean ± SEM channel open probability (P_o_) for currents recorded in control solution and currents recorded in the presence of DTT, DTNB and nisoldipine (Nisol) are shown below right. ^#,^*p < 0.05 vs Con ANOVA and Tukey’s Posthoc test.

The Ca_v_1.2 channel is expressed predominantly as two alternatively spliced variants in human heart. Exon 1a codes for the initial 46 amino acids in the N-terminus of the long N-terminal (NT) variant and exon 1b codes for 16 amino acids in the short NT variant (Fig. 2a). The short NT isoform also contains exon 45, a region that has been proposed to confer “oxygen sensing” properties to the channel^15^. We tested whether the two isoforms responded to thiol reducing and oxidising agents. The long NT isoform was exposed to the thiol reducing agent dithiothreitol (DTT) followed by the thiol oxidising agent dithio bis-nitrobenzoic acid (DTNB) in the same patch. In the absence of DTT or DTNB voltage step of the long NT isoform to 100 mV resulted in a P_o_ of 0.021 ± 0.001 (n = 11). When DTT was applied the P_o_ decreased on average to 0.008 ± 0.001 and then following addition of DTNB the P_o_ increased to 0.033 ± 0.002 (Fig. 2b). The magnitude of the current and the current-voltage relationship did not change (Fig. 2b), nor did the mean open dwell time (Table 1). The L-type Ca^2+^ channel antagonist nisoldipine attenuated the currents. Similar results were obtained when the short NT isoform was exposed to DTT and DTNB (n = 12; Fig. 2c). The current-voltage relationship was not altered by DTT or DTNB and nisoldipine attenuated the currents (Fig. 2c). The mean open dwell time was 21.01 ± 2.5 ms in DTT, 20.03 ± 3.6 ms in DTNB and this was not different from the open dwell time recorded in the absence of DTT or DTNB (n = 12; p = NS, Table 1). These data indicate that the short and long NT isoforms respond functionally to thiol modification and exon 1a and exon 45 do not encode the critical cysteines.

### Truncation of the C-terminus does not alter the response of the channel to thiol modification

**Figure 3:**
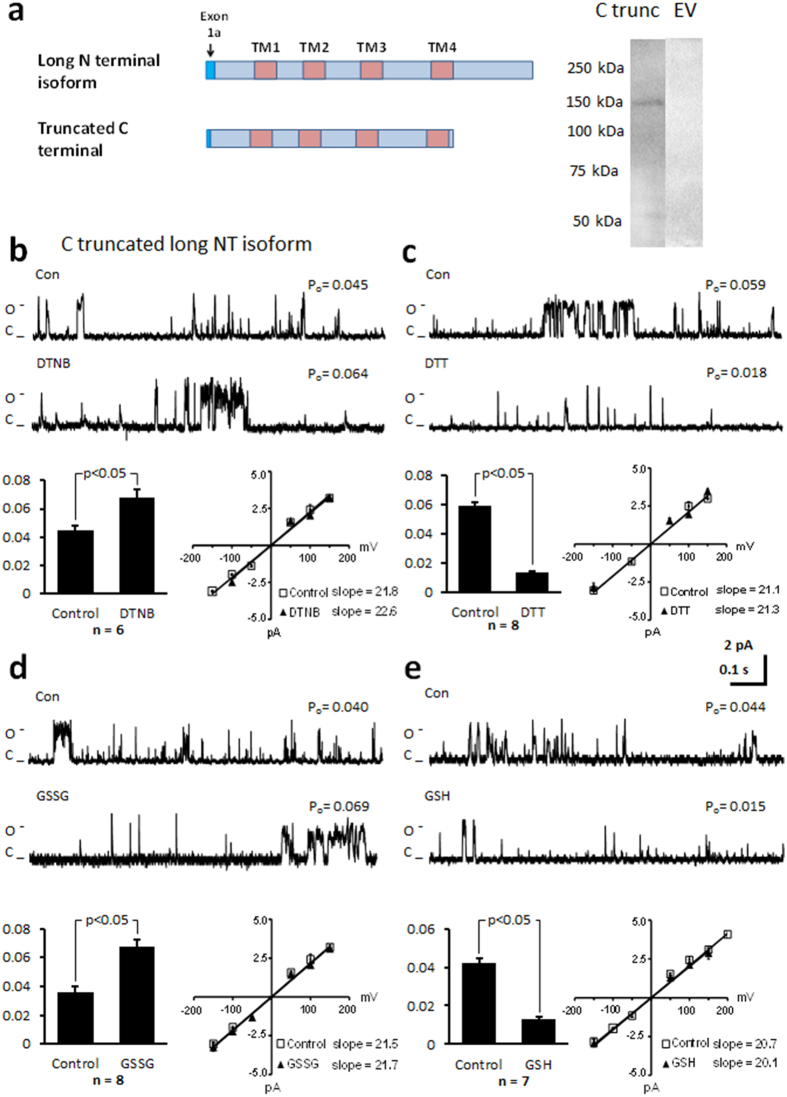
Truncating the C terminus of the long NT isoform significantly increases P_o_ but does not alter the response of the channel to thiol oxidising or reducing agents. (**a**) Schematic of full-length long NT isoform and long NT isoform indicating truncation of C terminus. Immunoblot of the truncated NT isoform of the Ca_v_1.2 channel protein indicated at approximately 150 kDa when probed with anti-Ca_v_1.2 antibody (C trunc) and protein from HEK293 cells expressing pcDNA3.1 plasmid minus Ca_v_1.2-His (Empty vector = EV). (**b**) Representative single-channel currents of the C truncated long NT isoform recorded at + 100 mV in the absence and presence of 200 μM DTNB in the same patch as shown. I–V relationship (below left) and the mean ± SEM channel open probability (P_o_) for currents recorded in control solution and currents recorded in the presence of DTNB are shown below right. (**c**) Representative single-channel currents of the C truncated long NT isoform recorded at + 100 mV in the absence and presence of 1 mM DTT in the same patch as shown. I–V relationship (below left) and the mean ± SEM channel open probability (P_o_) for currents recorded in control solution and currents recorded in the presence of DTT are shown below right. (**d**) Representative single-channel currents of the C-terminal truncated long NT isoform recorded at + 100 mV in the absence and presence of 2 mM GSSG in the same patch as shown. I–V relationship (below left) and the mean ± SEM channel open probability (P_o_) for currents recorded in control solution and currents recorded in the presence of GSSG are shown below right. (**e**) Representative single-channel currents of the C-terminal truncated long NT isoform recorded at + 100 mV in the absence and presence of 1 mM GSH in the same patch as shown. I–V relationship (below left) and the mean ± SEM channel open probability (P_o_) for currents recorded in control solution and currents recorded in the presence of GSH are shown below right.

To determine whether the C-terminus contained the critical cysteines, we engineered channel protein without the C-terminus by truncating the protein at leucine 1504 (Fig. 3a). Previous *in vitro* and *in vivo* studies have produced conflicting results with some groups reporting that the C-terminus imposes an inhibitory effect on channel currents and truncating the terminus results in a significant increase in current^28^,^29^,^30^, while others have argued that the C-terminus facilitates channel gating^31^. Upon voltage step to 100 mV we measured a P_o_ of 0.046 ± 0.016 (n = 12) for the C-terminal truncated channel. This is significantly larger than the P_o_ recorded in the long NT isoform (p < 0.05; Fig. 2). Following application of DTNB, the P_o_ further increased to 0.072 ± 0.018 (n = 6; Fig. 3b) and in separate experiments DTT decreased the P_o_ from 0.058 ± 0.002 to 0.013 ± 0.002 (Fig. 3c). There was no change in current-voltage relationship or mean open dwell time after application of DTNB but application of DTT significantly decreased mean open dwell time, presumably because the open probability of the channel was also decreased (Table 1). We then examined the effect of reduced glutathione (GSH) and oxidised glutathione (GSSG) on channel function. Exposure of the truncated protein to GSSG resulted in a significant increase in P_o_ (Fig. 3d), while exposure to GSH decreased P_o_ (Fig. 3e). No changes in current voltage relationship were recorded. However, mean open dwell time was decreased after application of GSH (Table 1). Our data confirm that truncation of the C-terminus appears to release an inhibitory effect of the C-terminal domain on channel gating but the C-terminus does not contain the functionally reactive cysteines.

### Mutation of cysteines in the N-terminus and loop III-IV linker region do not alter the response of the channel to thiol modification

**Figure 4:**
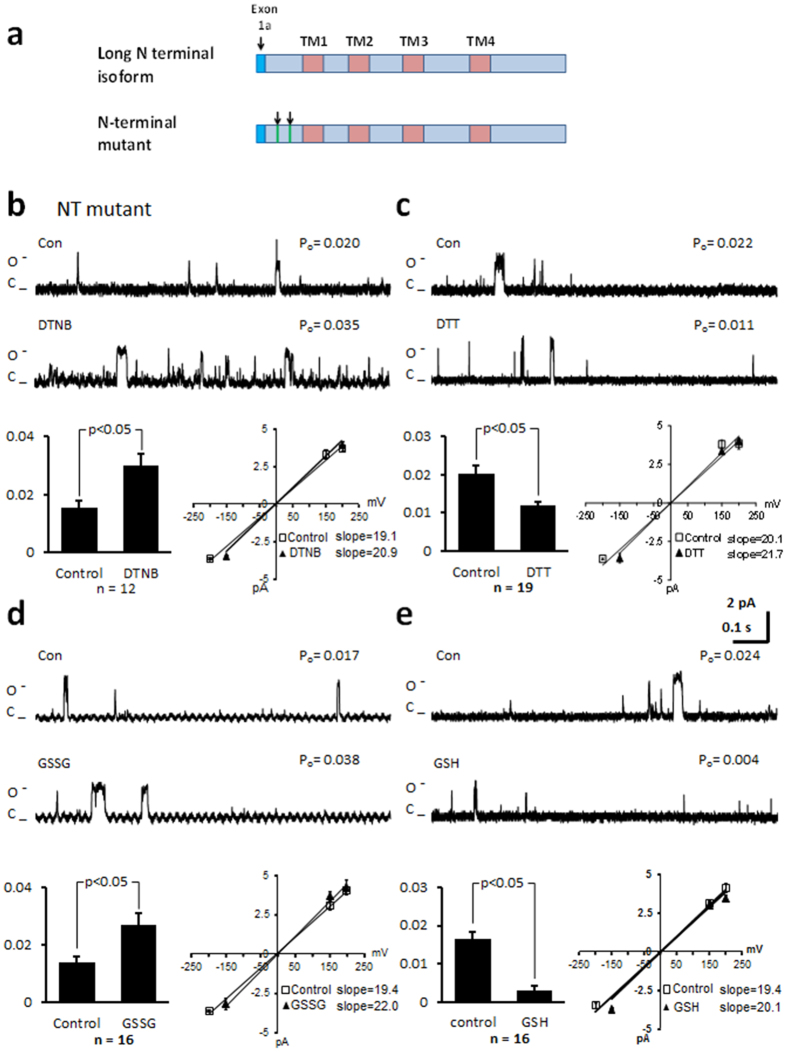
Mutation of cysteines in the N-terminus of the long NT isoform do not alter the response of the channel to thiol oxidising or reducing agents. (**a**) Schematic of the long NT isoform and long NT isoform indicating mutation of cysteines in the N-terminus (NT mutant). (**b**) Representative single-channel currents of the NT mutant recorded at + 100 mV in the absence and presence of 200 μM DTNB in the same patch as shown. I–V relationship (below left) and the mean ± SEM channel open probability (P_o_) for currents recorded in control solution and currents recorded in the presence of DTNB are shown below right. (**c**) Representative single-channel currents of the NT mutant recorded at + 100 mV in the absence and presence of 1 mM DTT in the same patch as shown. I–V relationship (below left) and the mean ± SEM channel open probability (P_o_) for currents recorded in control solution and currents recorded in the presence of DTT are shown below right. (**d**) Representative single-channel currents of the NT mutant recorded at + 100 mV in the absence and presence of 2 mM GSSG in the same patch as shown. I–V relationship (below left) and the mean ± SEM channel open probability (P_o_) for currents recorded in control solution and currents recorded in the presence of GSSG are shown below right. (**e**) Representative single-channel currents of the NT mutant recorded at + 100 mV in the absence and presence of 1 mM GSH in the same patch as shown. I–V relationship (below left) and the mean ± SEM channel open probability (P_o_) for currents recorded in control solution and currents recorded in the presence of GSH are shown below right.

**Figure 5:**
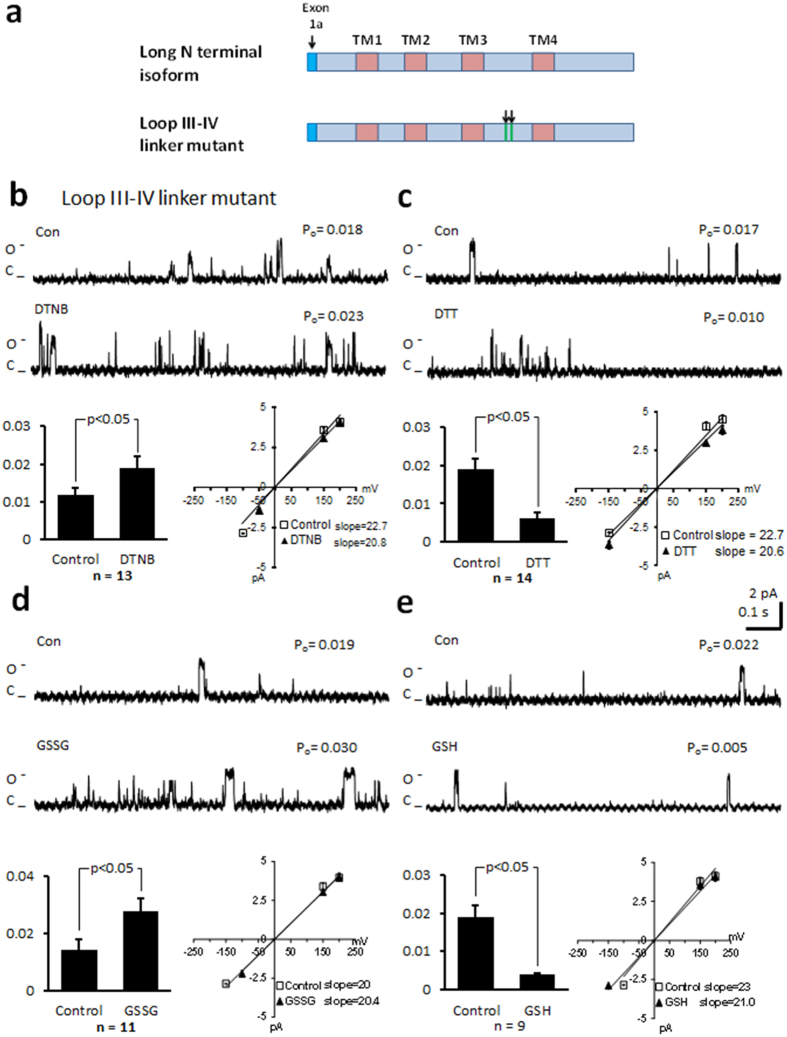
Mutation of cysteines in the cytoplasmic loop III-IV linker of the long NT isoform do not alter the response of the channel to thiol oxidising or reducing agents. (**a**) Schematic of the long NT isoform and long NT isoform indicating mutation of cysteines in the third cytoplasmic loop (loop III-IV linker mutant). (**b**) Representative single-channel currents of the loop III-IV mutant recorded at + 100 mV in the absence and presence of 200 μM DTNB in the same patch as shown. I–V relationship (below left) and the mean ± SEM channel open probability (P_o_) for currents recorded in control solution and currents recorded in the presence of DTNB are shown below right. (**c**) Representative single-channel currents of the loop III-IV mutant recorded at + 100 mV in the absence and presence of 1 mM DTT in the same patch as shown. I–V relationship (below left) and the mean ± SEM channel open probability (P_o_) for currents recorded in control solution and currents recorded in the presence of DTT are shown below right. (**d**) Representative single-channel currents of the loop III-IV mutant recorded at + 100 mV in the absence and presence of 2 mM GSSG in the same patch as shown. I–V relationship (below left) and the mean ± SEM channel open probability (P_o_) for currents recorded in control solution and currents recorded in the presence of GSSG are shown below right. (**e**) Representative single-channel currents of the loop III-IV mutant recorded at + 100 mV in the absence and presence of 1 mM GSH in the same patch as shown. I–V relationship (below left) and the mean ± SEM channel open probability (P_o_) for currents recorded in control solution and currents recorded in the presence of GSH are shown below right.

We predicted that cysteines within the transmembrane region are not likely to be reactive to thiol reduction or oxidation. Therefore, we mutated cysteines in the cytoplasmic regions. Mutation of cysteine 136 and 137 to alanine in the N-terminus of the long NT isoform (NT mutant, Fig. 4a) resulted in similar P_o_ values to the values measured in the *wt* long NT isoform (0.021 ± 0.003, n = 9; p = NS), when voltage was stepped to 100 mV. After application of DTNB, the P_o_ significantly increased from 0.017 ± 0.003 to 0.026 ± 0.003 (n = 12), and in separate experiments the P_o_ decreased from 0.020 ± 0.003 to 0.013 ± 0.002 after application of DTT (n = 19), without altering the current-voltage relationship or mean open dwell time (Table 1). Application of GSSG significantly increased P_o_ (Fig. 4d) while GSH significantly decreased P_o_ without altering the current-voltage relationship (Fig. 4e). Similar results were obtained when cysteines at 1210 and 1219 in the loop III-IV linker were mutated to serines (Fig. 5a). When the mutated channel protein was voltage stepped to 100 mV, P_o_ was similar to the *wt* long NT isoform (0.017 ± 0.003; n = 6; n = NS). Application of DTNB (Fig. 5b) or GSSG (Fig. 5d) increased P_o_ while DTT (Fig. 5c) and GSH (Fig. 5e) decreased P_o_ without altering the current-voltage relationship or the mean open dwell time (Table 1). These data indicate that the N-terminal cysteines and loop III-IV linker cysteines are not involved in functional modifications of the channel during changes in redox state.

### Mutation of cysteines in the loop I-II linker region attenuate the responses of the channel to thiol modification

**Figure 6:**
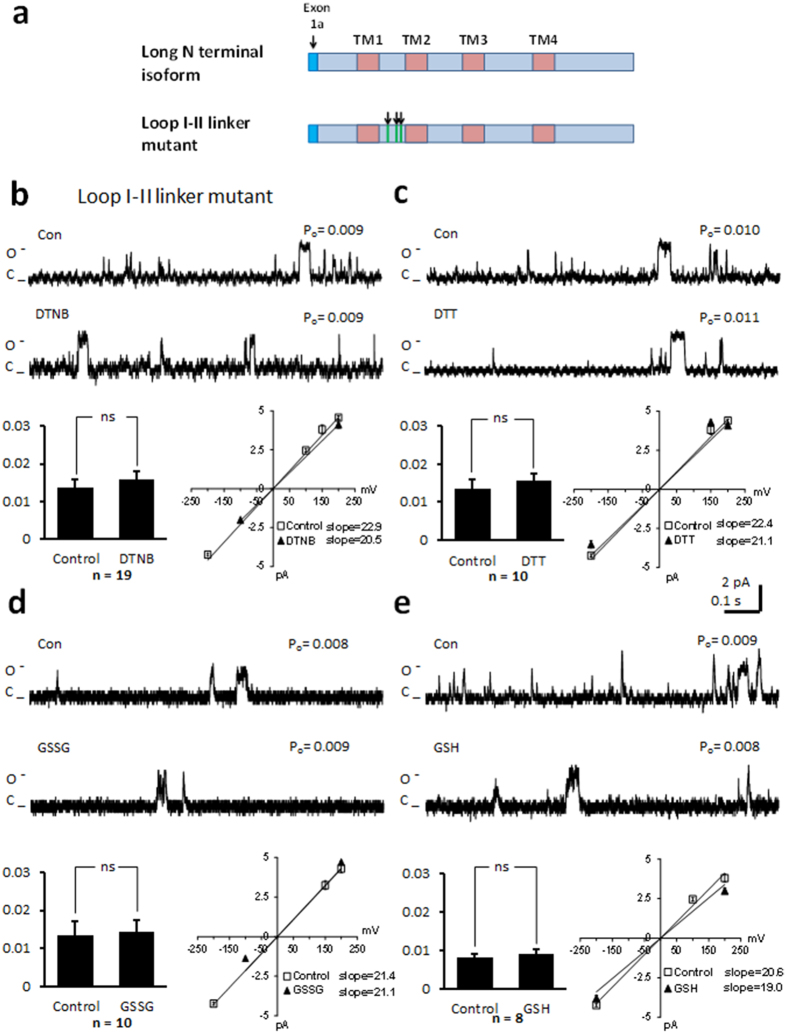
Mutation of cysteines in the loop I-II linker region of the long NT isoform attenuates the response of the channel to thiol oxidising or reducing agents. (**a**) Schematic of the long NT isoform and long NT isoform indicating mutation of 3 cysteines in the loop I-II region (loop I-II linker mutant). (**b**) Representative single-channel currents of the loop I-II mutant recorded at + 100 mV in the absence and presence of 200 μM DTNB in the same patch as shown. I–V relationship (below left) and the mean ± SEM channel open probability (P_o_) for currents recorded in control solution and currents recorded in the presence of DTNB are shown below right. (**c**) Representative single-channel currents of the loop I-II mutant recorded at + 100 mV in the absence and presence of 1 mM DTT in the same patch as shown. I–V relationship (below left) and the mean ± SEM channel open probability (P_o_) for currents recorded in control solution and currents recorded in the presence of DTT are shown below right. (**d**) Representative single-channel currents of the loop I-II mutant recorded at + 100 mV in the absence and presence of 2 mM GSSG in the same patch as shown. I–V relationship (below left) and the mean ± SEM channel open probability (P_o_) for currents recorded in control solution and currents recorded in the presence of GSSG are shown below right. (**e**) Representative single-channel currents of the loop I-II mutant recorded at + 100 mV in the absence and presence of 1 mM GSH in the same patch as shown. I–V relationship (below left) and the mean ± SEM channel open probability (Po) for currents recorded in control solution and currents recorded in the presence of GSH are shown below right.

**Figure 7:**
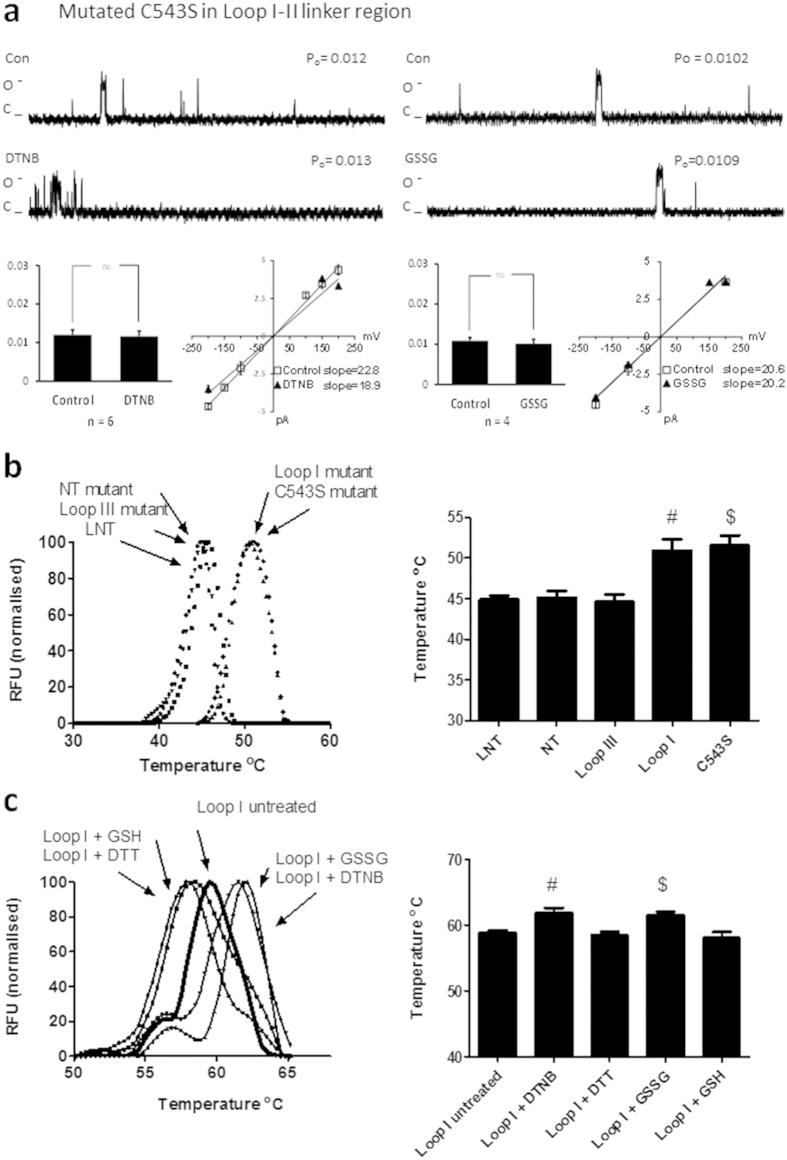
Mutation of cysteine 543 to serine alters the response of the channel to thiol oxidising or reducing agents and the thermal stability of the protein. (**a**) *Left* Representative single-channel currents of the C543S mutant recorded at + 100 mV in the absence and presence of 200 μM DTNB in the same patch as shown. I–V relationship (below left) and the mean ± SEM channel open probability (P_o_) for currents recorded in control solution and currents recorded in the presence of DTNB are shown below right. *Right* Representative single-channel currents of the C543S mutant recorded at + 100 mV in the absence and presence of 2 mM GSSG in the same patch as shown. I–V relationship (below left) and the mean ± SEM channel open probability (P_o_) for currents recorded in control solution and currents recorded in the presence of GSSG are shown below right (**b**) Thermal stability assays for full length long NT isoform (LNT) and mutant proteins. Fluorescence of the Sypro Orange increases as the proteins unfold and hydrophobic surfaces are exposed. Mean ± SEM of maximal temperatures for unfolding of LNT and mutant proteins as indicated at right. ^#^ p < 0.05 vs LNT, NT mutant and loop III-IV mutant proteins; ^$^p < 0.05 vs LNT, NT mutant and loop III-IV mutant proteins (**c**) Alterations in thermal stability for loop I-II peptides in the presence or absence of GSH, GSSG, DTT or DTNB. Fluorescence of the Sypro Orange increases as the proteins unfold and hydrophobic surfaces are exposed. Mean ± SEM of maximal temperatures for unfolding of loop as indicated at right. ^#^ p < 0.05 vs loop I-II + DTT; ^$^ p < 0.05 vs loop I-II + GSH. ANOVA and Tukey’s posthoc test.

We mutated cysteine 519 and 543 to serine, and cysteine 547 to alanine in the cytoplasmic loop between domains I and II (Fig. 6a). This region is responsible for binding of the auxiliary beta subunit^8^. In the absence of thiol modifying agents, channel function was altered. After voltage stepping to 100 mV we recorded a P_o_ of 0.013 ± 0.002 (n = 19; Fig. 6b). This was significantly less than the P_o_ recorded in the *wt* long NT isoform (0.021 ± 0.003, n = 9; p < 0.05; Fig. 2), and suggests that mutation of these cysteines altered the gating of the channel protein such that it was less likely to open. Application of DTNB, DTT, GSSG or GSH had no effect on the P_o_ or current-voltage relationship (Fig. 6b–e). Mean open dwell time was also unchanged under the various treatments (Table 1). Finally we find that mutation of cysteine 534 to a serine is sufficient to alter P_o_ in the absence of thiol modifying agents (P_o_ 0.012 ± 0.001; n = 6) and attenuate the effects of GSH and GSSG on P_o_ (Fig. 7a).

### Mutation of cysteines in the loop I-II linker region alters protein folding

We performed *in vitro* thermal stability assays on each of the full length mutant proteins and the *wt* long NT isoform. Fluorescence of the Sypro Orange increases as the protein unfolds and hydrophobic surfaces are exposed. At higher temperatures the fluorescence decreases as the unfolded protein starts to aggregate. The maximal fluorescence was determined as the melting temperature of the protein. The *wt* long NT isoform, the NT mutant and loop III-IV linker mutant proteins all demonstrated similar melting peak profiles (Fig. 7). However, the loop I-II linker mutant protein and C543S mutant protein unfolded later and peaked at a higher temperature indicating that the stability of the protein was altered. This may have accounted for the difficulty in opening the channel (the lower P_o_) recorded in the loop I-II linker mutant protein and C543S mutant protein under control conditions (Figs 6 and 7b), compared with the P_o_ recorded for the long NT isoform under control conditions (Fig. 2). We constructed the cytoplasmic loop I-II linker region as a 118 amino acid peptide and exposed the peptide to DTT, DTNB, GSH or GSSG and assessed the melting profiles. Exposure to thiol oxidising agents resulted in the unfolding of the loop I-II linker peptide at a significantly higher temperature than when the peptide was exposed to DTT or GSH (Fig. 7c). These data indicate that the loop I-II linker region contains cysteines that are responsible for altering structure and function of the Ca_v_1.2 protein during changes in redox state. Cysteine 543 appears to play a pivotal role.

## Discussion

In this study we examined whether direct modification of thiol groups on the Ca_v_1.2 channel protein is sufficient to alter function. We find that mutation of cysteines at 519, 543 and 547 attenuates the effects of thiol modifying agents on channel function and that C543 is a critical cysteine. As a result, we clarify three areas concerning regulation of Ca_v_1.2 channel protein. Firstly, by identifying the cysteine that is responsive to thiol modification we confirm that a regulatory protein does not appear to be necessary to regulate channel function. We expressed the human long NT isoform of the Ca_v_1.2 channel in HEK293 cells and purified the channel protein. The protein forms a functional ion-conducting channel when reconstituted in liposomes assessed as single channel currents using patch-clamp technique. We find that outward currents can be elicited when driving voltage in the positive direction and inward currents are elicited when driving the voltage in the negative direction, consistent with symmetric conditions (Fig. 2c). Therefore, the channel allows the movement of ions in either direction. The technique allows for the measurement of changes in channel function as a result of direct modification of the channel protein. This is superior to studying changes in function of ion channels in expression systems such as HEK293 cells or xenopus oocytes because channel function will be influenced by regulatory proteins present in the cell. After application of thiol reducing agents DTT or reduced glutathione, the P_o_ of long NT isoform of Ca_v_1.2 decreased without altering the magnitude of the current or the mean open dwell time (Fig. 2). This is consistent with reports in native L-type Ca^2+^ channel currents that demonstrate an inhibition of peak inward current after application of DTT without altering the current-voltage relationship^13^,^14^,^17^,^32^. Conversely, application of DTNB or GSSG increased the P_o_ without altering the magnitude of the current or the mean open dwell time (Fig. 2), similar to effects recorded in native L-type Ca^2+^ channel currents^13^,^14^,^17^,^32^. At least in cardiac myocytes, the native channel appears to respond to changes in thiol group redox state similar to the purified Ca_v_1.2 channel protein.

The second point that we clarify concerns whether the channel has an intrinsic ability to sense changes in oxygen tension^33^. It has been proposed that the short NT isoform contains an “oxygen sensing region” encoded by exon 45^15^. We have previously demonstrated that hypoxia does not directly alter the function of the long NT isoform or the short NT isoform reconstituted in proteoliposomes^16^. If this is true, the channel must be responding as a “redox sensor”. In this study we confirm that the long and short NT isoforms respond to changes in thiol redox state. Hypoxia (lowering PO_2_ to 15 mm Hg) decreases peak inward native L-type Ca^2+^ channel current (I_Ca-_L) in cells and application of thiol oxidising agents attenuates the effect^13^,^14^,^17^. A number of sources of reactive oxygen species have been described in the heart including superoxide generated from NAD(P)H-oxidase and xanthine oxidase^34^. The mitochondrial electron transport chain is a source of superoxide production by reducing molecular oxygen to superoxide anions^33^. Superoxide is rapidly dismutated to hydrogen peroxide that can readily cross membranes to react with thiol groups^5^. Mitochondrial superoxide is decreased during hypoxia^35^,^36^,^37^,^38^ and catalase (that catalyses the conversion of hydrogen peroxide to water and oxygen) mimics the effects of hypoxia on native I_Ca-L_ function^17^. Therefore, our data are consistent with the channel behaving as a “redox sensor”. That is, thiol groups on the channel protein respond to alterations in cellular redox state rather than the channel behaving as an “oxygen sensor”.

The third point that we clarify is the manner in which the C-terminus influences channel gating. We truncated the C-terminus and reconstituted the truncated channel protein in liposomes to measure function. The C-terminus is proposed to contain the sites for calcium dependent inactivation of current and phosphorylation by CAMKII and other serine/threonine kinases^39^. It is also suggested that the C-terminus imposes an inhibitory regulation of the current^28^,^29^,^30^ but *in vivo* studies have argued against this^31^. We find that truncation of the C-terminus results in a significant increase in P_o_ (Fig. 3), consistent with the C-terminus imposing an inhibitory effect on channel function.

The structure of the full length Ca_v_1.2 channel protein is not known. Our results suggest that the alpha interacting domain plays a significant role in regulating channel opening. We find that P_o_ is significantly decreased under basal conditions (Fig. 6). In addition, mutation of cysteines within the loop I-II linker region resulted in an attenuation of the effect of thiol modifying agents on function. Finally we find that mutation of C543S is sufficient to attenuate the effect of thiol modifying agents. The melting point for folding of the channel was also altered in the loop I-II linker mutant suggesting that the I-II region may be important for maintaining protein structure (Fig. 7). Although the melting point was altered, the I-II linker mutant and C543S mutant proteins remained functionally active when voltage stepped in proteoliposomes (Figs 6 and 7) indicating the cysteine at 543 is important for conferring sensitivity to thiol modification.

Although this technique enables direct functional assessment of the Ca_v_1.2 subunit, the alpha subunit does not function in isolation from the auxiliary α_2_δ and β_2_ subunits in the native heterotetrameric protein. Since the β_2_ subunit modifies inactivation kinetics of the current and the cysteine at 543 lies within the alpha interacting domain where the β_2_ subunit binds to the alpha subunit, redox modification of the Ca_v_1.2 subunit could alter the behaviour of the β_2_ subunit. In addition direct redox modification of the auxiliary subunits could modify the response of the native channel *in vivo*. Reactive oxygen species can alter the activity of many proteins including signaling proteins such as serine threonine kinases or tyrosine kinases that can further influence the function of Ca_v_1.2 subunit.

We conclude that the function of the Ca_v_1.2 channel protein can be directly regulated by thiol reducing or oxidising agents including glutathionylation. We have identified the critical cysteine for the response. Our data suggest that *in vivo* auxiliary or regulatory proteins modulate channel function in addition to direct regulation. We propose that under conditions of changes in cellular redox state (hypoxia or oxidative stress), direct regulation of the Ca_v_1.2 alpha subunit is sufficient to alter calcium influx. Since glutathionylation of Ca_v_1.2 occurs in the ischemic human heart^25^, and increased calcium influx through Ca_v_1.2 is sufficient to induce hypertrophic growth^23^,^40^ direct regulation of Ca_v_1.2 channel protein during reperfusion may contribute to the pathology associated with ischemic heart disease.

## Methods

### Preparation of mutant constructs

The cDNA of the long N terminal isoform of the Ca_v_1.2 was cloned into pcDNA3.1 vector (Invitrogen), and modified to include a HIS_6_ tag at the N-terminus. This construct was used as the backbone for mutagenesis. Site-directed mutagenesis was undertaken to make point mutations in the LNT cDNA using the Quikchange kit according to the manufacturers protocol (Stratagene). Cysteines were mutated to serines. In some cases cysteines were mutated to alanine in order to prevent potential phosphorylation of the –OH group *in vitro* when preceded by an arginine at –3 position. Two oligonucleotide primers containing each desired mutation were extended by *Pfu Ultra II DNA polymerase* (Stratagene) during PCR. A standard PCR reaction was prepared with 1 μl of cDNA; 1μl of 10 pmol forward and reverse primers each, 5 μl of 10 XPfu buffer; 2 μl of 5 mM dNTP’s; 1 μl of *Pfu Ultra II DNA polymerase* and 39 μl of nuclease free water. A standard amplification protocol was performed (MJ Research PTC-200 Thermal Cycler PCR). Mutants were confirmed by automated DNA sequencing and restriction digests using *XbaI* and *BamHI* restriction enzymes (Promega). Correct mutant DNA was then purified from an overnight culture using low copy plasmid purification protocol (Nucleobond Xtra Midi plus) kit.

### Preparation of cytoplasmic loop 1-II linker construct

The cDNA of the truncated C-terminal construct of the Ca_v_1.2 channel was used as a template to amplify the linker I-II region using an appropriate primer. The primer was designed to introduce *BamHI* and *EcoRI* restriction sites in the cDNA. A standard reaction mix was prepared with using KOD Hot Start DNA polymerase kit as per manufacturer’s instructions (Merck-Millipore). The linker I-II region sequence incorporating Glycine 377 to asparagine 495 was cloned into *BamHI* and *EcoRI* sites in pGEX-2T vector with a glutathione-S-transferase (GST) tag. Vector and insert were digested with *BamHI* and *EcoRI* restriction enzymes at 37 °C for 1 h. Vector and insert were gel purified and then ligated at 4:1 ratio using T4 DNA ligase (Invitrogen). Ligated product was transformed into XL-2 blue competent cells. Colony PCR was performed to identify the colonies containing the insert. Positive clones were further confirmed by restriction digests using *BamHI* and *EcoRI* as well as by Sanger DNA sequencing. The recombinant pGEX-2T vector was grown on Luria Bertini-Ampicillin-Chloramphenicol (LB-Amp-Chloramp) agar plates overnight. Several colonies were then inoculated in 5 ml LB-Amp-Chloramp broth at 225 rpm, 37 °C for approximately 3 h. Once the OD_600_ reached 0.6, 250 μl of 100 mM IPTG was added to the culture and then incubated for approximately 5 h at 37 °C. Following incubation, the cells were centrifuged at 20000 rcf for 5 min at room temperature. The cell pellet was resuspended in 1× phosphate buffered saline (PBS) containing 1× protease inhibitor tablet (Complete Mini Protease inhibitor, Roche). The cell suspension was then lysed by sonication and clarified by centrifugation at 20000 rcf for 5 min at 4 °C.

### Expression and purification of Ca_v_1.2

HEK293 cells (5 × 10^6 ^cells per l5 cm plate) were transfected with 15μg plasmid DNA using 35μl Lipofectamine transfection reagent according to the manufacturers protocol (Invitrogen). Cells were harvested 48 h after transfection. The cell pellet was lysed using lysis buffer (50 mM NaH_2_PO_4_.2H_2_O, 300 mM NaCl, 10 mM Imidazole and 1% Tween-20, pH 8.0). The cell suspension in the lysis buffer was sonicated and centrifuged at 10000 rpm for 10 min at 4 °C before the addition of Ni-NTA agarose beads (Qiagen). The lysate was placed on a magnetic stand and the beads were allowed to migrate towards the magnet, after which the supernatant was removed, and the beads were washed 3–4 times with 500μl wash buffer (50 mM NaH_2_PO_4_.2H_2_O, 300 mM NaCl, 20 mM Imidazole and 0.05% Tween-20, pH 8.0). After the last wash, 30μl of elution buffer (50 mM NaH_2_PO_4_.2H_2_O, 300 mM NaCl, 250 mM Imidazole and 0.05% Tween 20, pH 8.0) was added to the beads and was incubated for 1 min at room temperature The beads were allowed to migrate to the magnetic stand, following which the supernatant was removed and stored at –80 °C for later use. The purified protein was analyzed by SDS-PAGE and immunoblotting.

### Preparation of proteoliposomes

The purified Ca_v_1.2 channel protein was reconstituted into liposomes using dehydration/ rehydration method^41^. Phosphatidylcholine was dissolved in chloroform and was dried under high nitrogen gas. The lipid was reconstituted in dehydration/rehydration buffer (200 mM NaCl and 5 mM HEPES, pH 7.4) and then, was sonicated for 5 min. The purified channel protein was incubated with the lipid for 1 h at room temperature on an end over end shaker, following which 10 mg Biobeads (Bio-Rad) was added to the suspension and was incubated for 2 h at room temperature. The suspension was then centrifuged at 90000 rpm for 30 min at 4 °C. The proteoliposome pellet was resuspended in dehydration/rehydration buffer and was dehydrated at 4 °C for 4 hr. Each proteoliposome spot was rehydrated for 48 h at 4 °C with dehydration/rehydration buffer.

### Single channel patch recordings

A small aliquot of rehydrated proteoliposomes was placed in the patch-clamp bath filled with liposome loading solution (200 mM NaCl, 20 mM BaCl_2_ and 5 mM HEPES, pH 7.4) to promote the blister formation of liposomes for 10 min at room temperature. The loading solution was then replaced by recording solution (100 mM BaCl_2_, 50 mM NaCl and 10 mM HEPES, pH 7.4) to facilitate single channel current recording. Functional examination of Ca_v_1.2 channel protein reconstituted in liposomes was performed using the patch clamp technique. External and pipette solution contained recording solution with 2μM BayK8644 (Sigma) to enhance channel opening. The back filled microelectrode with pipette solution had an average resistance of 16–17 MΩ. A gigaohm ohm seal was achieved upon contact between the microelectrode and a liposome blister. Single channel currents were filtered at 1 kHz, digitized at 100 kHz, and analysed using pClamp software (Molecular devices). The Ca_v_1.2 channel was determined by the magnitude of the current, changes in open probability of the channel (P_o_) and sensitivity of the current to the L-type calcium channel antagonist nisoldipine. The recordings were made for a duration of 10 min. Clampfit 10.5 software was used for analysing the total number of events that the channel was open. The amplitude distribution of single channel events was analysed by Gaussian function and was fitted with Levenberg-Marquardt method (see Fig. 1C). The mean open time of the channel was determined by constructing frequency histograms of open times obtained from single channel recording.

### Immunoblotting of Ca_v_1.2 protein

The purified channel protein (50μg) was incubated with 5× sample buffer (0.125 M Tris-HCl, pH 6.8, 4% SDS, 20% glycerol, 15% β-mercaptoethanol and 0.01% bromophenol blue) for 30 min at 45 °C. The channel proteins were separated by 6% sodium dodecyl sulphate – polyacrylamide gel electrophoresis (SDS-PAGE) for 1.5 h at 25 mA constant current per gel. The channel protein was electrophoretically transferred from the SDS-PAGE gel to nitrocellulose membrane using a wet transfer apparatus (Mini-Trans blot, Bio-Rad) in transfer buffer (40 mM Tris-HCl pH 7.4, 20 mM Sodium Acetate, 2 mM EDTA, 0.1% SDS) for 16 h at 30 V, 400 mA and 100 W with constant voltage at 4 °C. After transfer, the membrane was incubated in blocking buffer (5% bovine serum albumin (BSA), 0.05% Tween-20 in PBS, pH 7.4) for 1 h at room temperature. The membrane was then probed with an anti Ca_v_1.2 channel primary antibody (anti-Cav pan-α_1c_ subunit, Alomone labs) and then a goat anti-rabbit HRP conjugate secondary antibody (Abcam). The channel protein was finally, detected by (Luminata Forte, Western HRP substrate, Millipore).

### Thermal stability assay

Wild type and mutant channel proteins were expressed individually in HEK293 cells. The protein (50μg) was incubated with 5x reduced sample buffer (0.3125 M of 0.5 M Tris HCl pH 6.8; 10% SDS; 0.5 M DTT; 50% glycerol; 0.01% bromophenol blue) for 5 min at 95 °C. The channel protein was separated by 6% SDS-PAGE for 1.5 h at 25 mA constant current per gel. The 240–245 kDa protein band corresponding to the molecular weight marker was excised from the gel and was cut into fine pieces. The fine pieces of gel were immersed in 0.5 ml of elution buffer (50 mM Tris-HCl, 150 mM NaCl and 0.1 mM EDTA pH 7.5) and incubated overnight in a rotary shaker at room temperature. Following incubation, the suspension was centrifuged at 10000 × g for 10 min at 20 °C. The supernatant was transferred into a new tube. The supernatant was also tested for the presence of protein by SDS-PAGE. The purified protein (25μg) was mixed with 1:1000 dilution of 5000X Sypro Orange (Molecular Probes). All experiments were performed in triplicates. The samples were heated from 25 °C to 95 °C with a heating rate of 1 °C/min. Protein thermal unfolding curves were monitored by detection of changes in fluorescence of the Sypro Orange dye. The melting temperature (Tm) of wild type and mutant proteins was determined by converting the raw fluorescence data to the negative first derivative of the fluorescence with respect to the temperature (–dF/–dT).

### Statistical analysis

Results are reported as mean  ±  SEM here indicated. Statistical comparisons of responses between unpaired data were made using the student’s *t*-test or between groups of data using one-way ANOVA and the Tukey’s posthoc test.

## Additional Information

**How to cite this article**: Muralidharan, P. *et al*. Evidence for redox sensing by a human cardiac calcium channel. *Sci. Rep*. **6**, 19067; doi: 10.1038/srep19067 (2016).

## Figures and Tables

**Table 1:** Dwell times of currents recorded in *wt* and mutant protein.

	LNT	SNT	C trunc	NT	Loop III-IV	Loop I-II
Control	18.6 ± 2.4 (13)	19.1 ± 2.3 (12)	22.34 ± 2.5(6)	17.44 ± 1.1(8)	17.03 ± 1.1(5)	20.9 ± 0.6(11)
DTT	19.06 ± 2.7(11)	21.01 ± 2.5(12)	11.3 ± 1.8(6)*	16.57 ± 1.7(8)	16.5 ± 1.5(6)	21.1 ± 2.4(6)
DTNB	20.02 ± 2.6(11)	20.03 ± 3.6(12)	23.3 ± 0.8(6)	16.7 ± 1.7(8)	17.8 ± 1.8(5)	21.1 ± 2.4(11)
GSH	21.1 ± 1.5(5)	19.2 ± 2.5(6)	14.2 ± 1.7(9)*	17.2 ± 2.6 (9)	17.0 ± 1.1(5)	20.5 ± 2.1(4)
GSSG	20.4 ± 0.4(5)	20.3 ± 2.3(6)	23.1 ± 1.3(7)	18.6 ± 2.4(9)	17.6 ± 1.2 (7)	19.5 ± 1.9(5)

Mean ± SEM of dwell times (ms) of current recorded in long NT isoform (LNT), short NT isoform (SNT), C truncated long NT isoform (C trunc), NT mutant (NT), Loop III-IV linker mutant (Loop III-IV) or Loop I-II linker mutant (Loop I-II) under control conditions (absence of thiol modifying agent), in the presence of 1 mM DTT, 200 μM DTNB, 1 mM GSH or 2 mM GSSG. The number of experiments performed is indicated in parentheses. *p < 0.05 vs C trunc Control ANOVA and Tukey’s Posthoc test.
